# Supplementary figures and images for: Extending density surface models to include multiple and double-observer survey data
Source: PeerJ. 2021 Sep 2;9:e12113. doi: 10.7717/peerj.12113 (PMC8418794; doi:10.7717/peerj.12113)

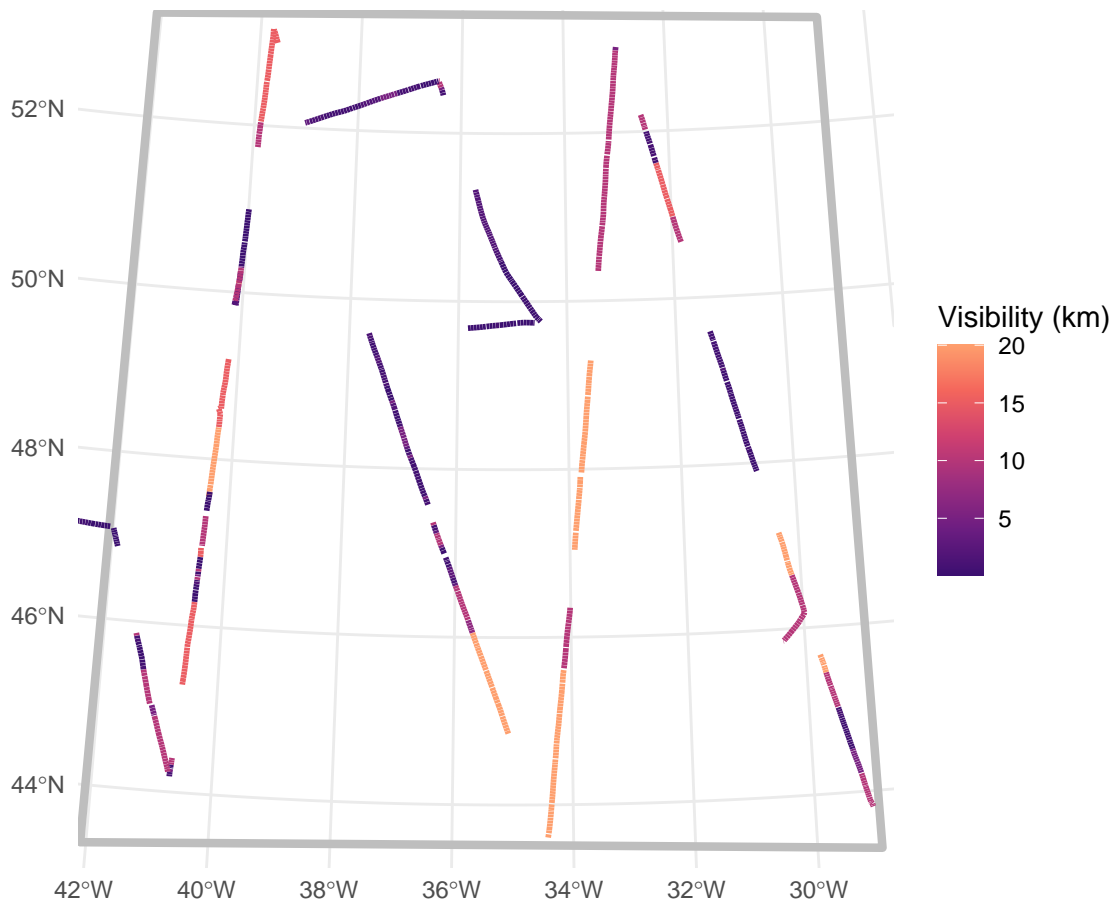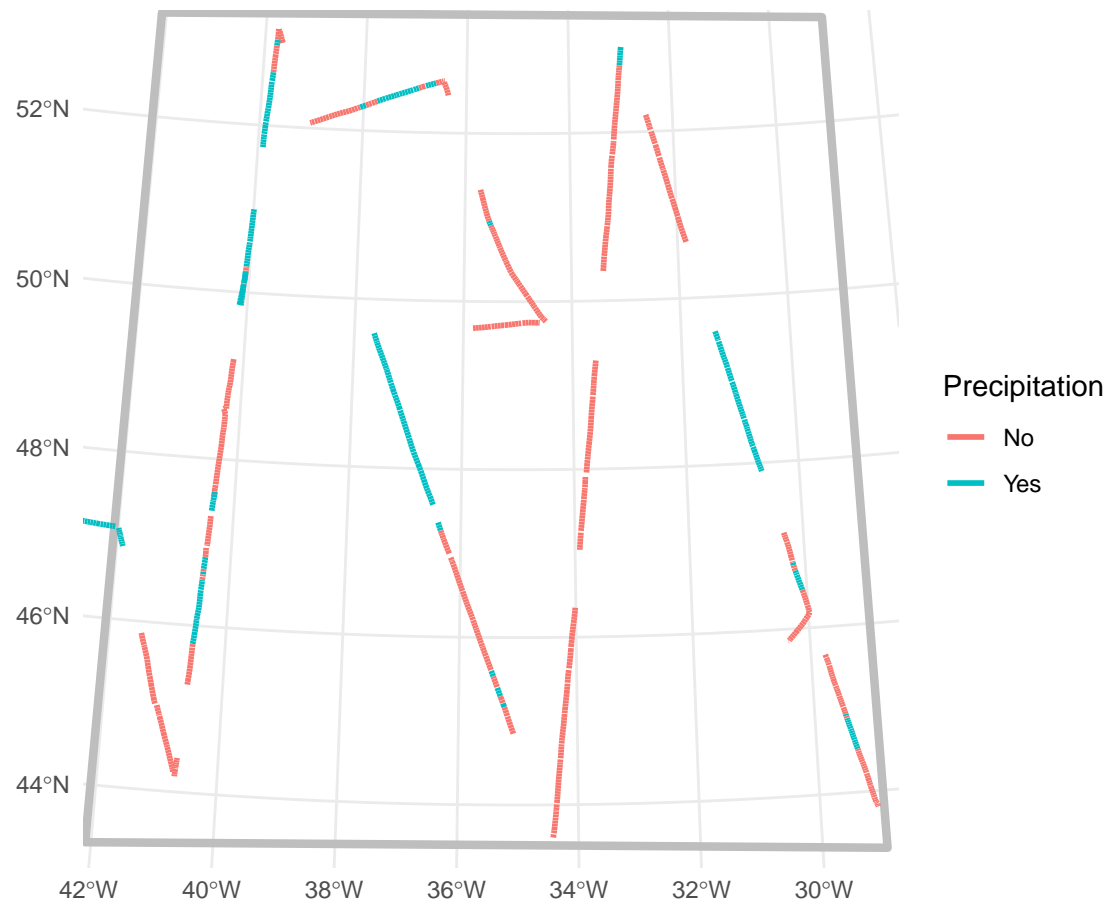

Supplement: Supplemental Information 1 — Position of the fulmar study area (thick grey box) within the North Atlantic, with bathymetry (coloured lines) and land (grey polygons) for reference. Transects are shown as the thicker black lines, note that only data from those within the study area were used in our analysis. [file peerj-09-12113-s001.pdf]

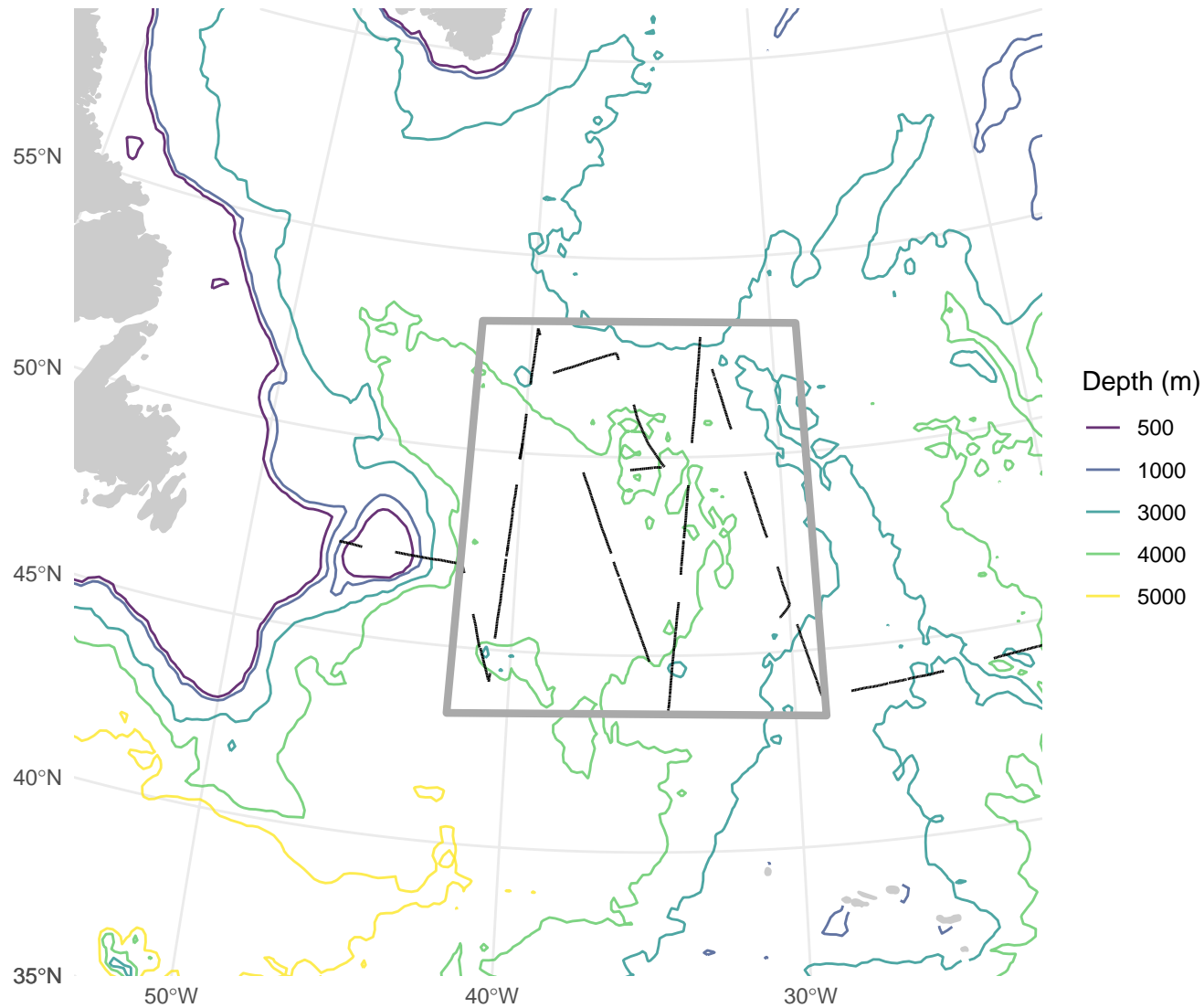

Supplement: Supplemental Information 2 — Lines indicate transect positions, thick grey box the study area as previously. [file peerj-09-12113-s002.pdf]

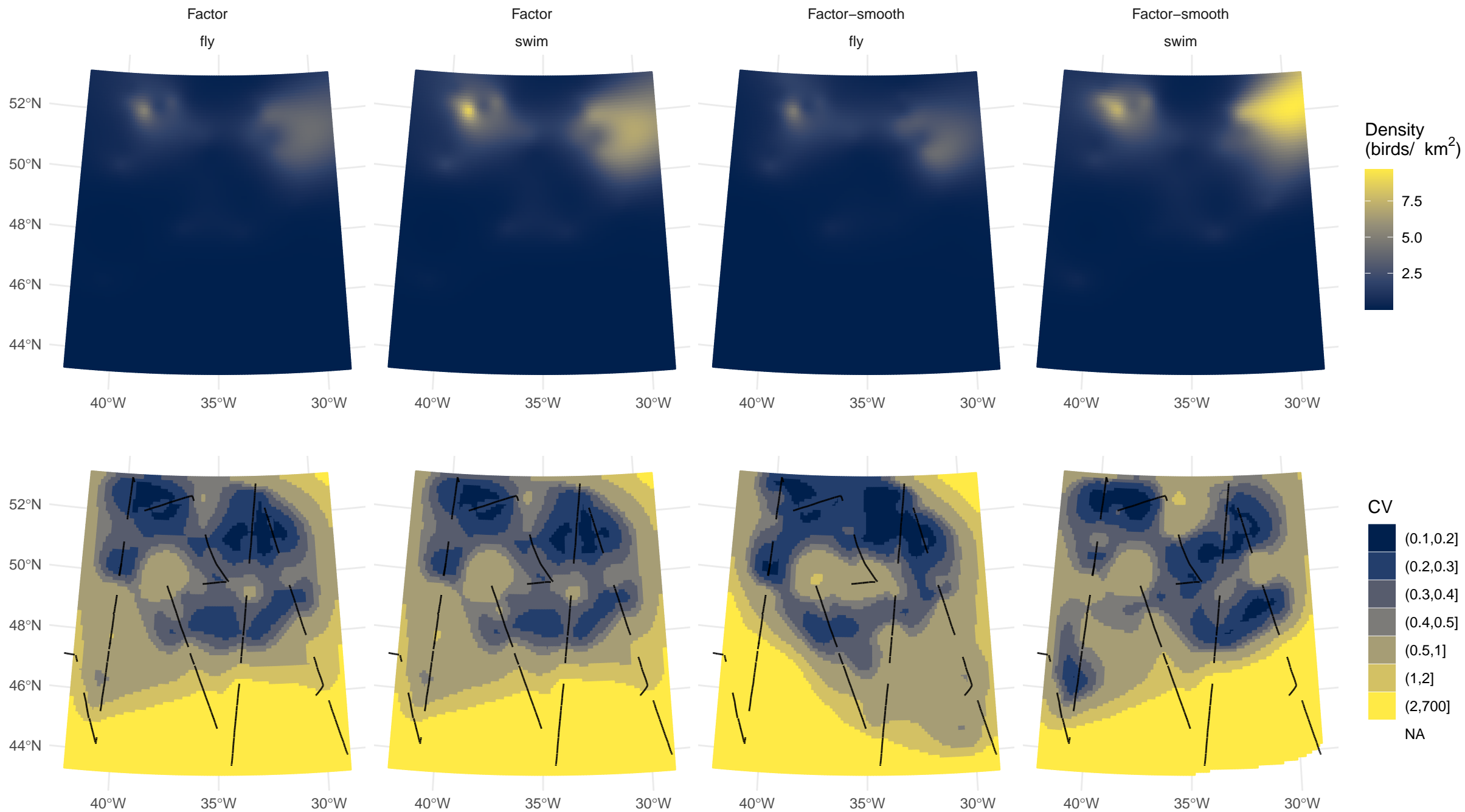

Supplement: Supplemental Information 3 — Predictions (top row) and coefficients of variation (bottom row) by platform and model (columns). The factor model (model (3) is used in the left two columns and factor-smooth model (model (4)) in the right two columns. The black lines show the survey segments. Compared to Fig. 4 we see differences in the predictions and the uncertainty. As the model formulation dictates, platform differences in the factor model dictate the overall level of the plot, whereas the factor-smooth models vary in their pattern too. This is particularly notable for the uncertainty plots. [file peerj-09-12113-s003.pdf]
